# Supplementary material for: The Thyroid Hormone Axis and Female Reproduction
Source: Int J Mol Sci. 2023 Jun 6;24(12):9815. doi: 10.3390/ijms24129815 (PMC10298303; doi:10.3390/ijms24129815)
Supplement: Supplementary file 1 [file ijms-24-09815-s001.zip › ijms-2404875-File S1.pdf]

Search Strategy

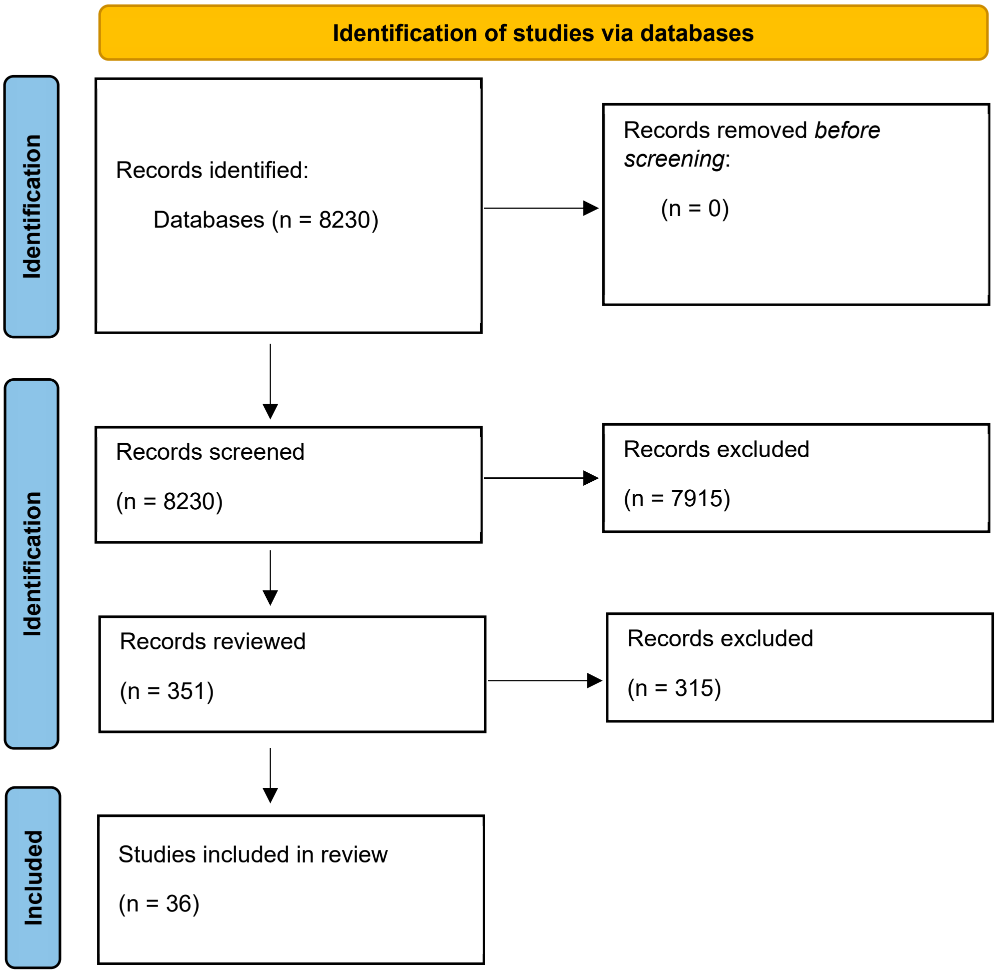

Pubmed, Embase, and Web of Science searches were performed and records subsequently excluded to retain only systematic reviews, guidelines, and randomized controlled trials. These studies were assessed for relevant information and subsequent branching searches were performed to ensure comprehensive inclusion of all relevant information.

PubMed

("Thyroid Gland"[Mesh] OR "Thyroid Diseases"[Mesh] OR "Thyroid Carcinoma, Anaplastic"[Mesh] OR "Receptors, Thyroid Hormone"[Mesh] OR "Thyroid Function Tests"[Mesh] OR "Thyroid Hormones"[Mesh] OR "Antithyroid Agents"[Mesh] OR Thyroid[tiab] OR goiter[tiab] OR hyperthyroid*[tiab] OR hypothyroid*[tiab] OR dysthyroid*[tiab] OR graves disease[tiab] OR grave's disease[tiab] OR graves' disease[tiab] OR Basedow Disease[tiab] OR basedows' disease[tiab] OR basedow's disease[tiab] OR (Hashimoto*[tiab] AND (Struma[tiab] OR syndrome[tiab] OR disease[tiab])) OR thyrotoxicosis[tiab] OR hyperthyroxinemia[tiab] OR myxedema[tiab] OR thyroiditis[tiab] OR ths deficien*[tiab] OR calcitonin[tiab] OR dextrothyroxine[tiab] OR diiodotyrosine[tiab] OR monoiodotyrosine[tiab] OR thyronines[tiab] OR thyroxine[tiab] OR triiodothyronine[tiab] OR thyroidectomy[tiab] OR Thyroidectomies[tiab] OR t3 receptor*[tiab] OR t4 receptor*[tiab] OR DIT receptor*[tiab] OR MIT receptor*[tiab] OR T3 test*[tiab] OR

t4 test*[tiab] OR tsh test*[tiab] OR thyroglobulin antibody[tiab] OR antithyroid*[tiab] OR anti-thyroid*[tiab] OR euthyroid[tiab]) AND ("Reproductive Physiological Phenomena"[Mesh] OR "Genitalia, Female"[Mesh] OR "Female Urogenital Diseases and Pregnancy Complications"[Mesh] OR "Infant, Low Birth Weight"[Mesh] OR "Pregnant Women"[Mesh] OR "Abortion Applicants"[Mesh] OR reproduction[tiab] OR reproductive[tiab] OR sexual[tiab] OR intercourse[tiab] OR conception*[tiab] OR preconception*[tiab] OR periconception*[tiab] OR contraceptive*[tiab] OR uterus[tiab] OR uteri[tiab] OR uterine[tiab] OR ovary[tiab] OR ovaries[tiab] OR ovarian[tiab] OR oocyte*[tiab] OR oogenesis[tiab] OR embryo*[tiab] OR endometri*[tiab] OR PCOS[tiab] OR pregnan*[tiab] OR obstetric[tiab] OR obstetrical[tiab] OR gynecol*[tiab] OR antenatal[tiab] OR prenatal[tiab] OR childbirth[tiab] OR child birth[tiab] OR childbearing[tiab] OR labor[tiab] OR labour[tiab] OR parturition[tiab] OR delivery outcome*[tiab] OR menarche[tiab] OR amenorrhea[tiab] OR gametogenesis[tiab] OR ovulat*[tiab] OR menstrual[tiab] OR menstruat*[tiab] OR menopaus*[tiab] OR premenopaus*[tiab] OR gestation*[tiab] OR gravid*[tiab] OR fertility[tiab] OR fertile[tiab] OR infertility[tiab] OR infertile[tiab] OR subfertility[tiab] OR subfertile[tiab] OR fecundity[tiab] OR fertilization[tiab] OR stillbirth*[tiab] OR stillborn*[tiab] OR miscarriage*[tiab] OR abortion*[tiab] OR fetal loss*[tiab] OR fetal death*[tiab] OR low birth weight*[tiab] OR low birthweight*[tiab] OR small for gestational age[tiab] OR SGA[tiab] OR growth retardation[tiab] OR growth restriction[tiab] OR restricted growth[tiab] OR IUGR[tiab] OR preterm birth*[tiab] OR pre-term birth*[tiab] OR premature birth*[tiab] OR premature labor[tiab] OR preterm labor[tiab] OR preterm deliver*[tiab] OR premature deliver*[tiab] OR preterm infant*[tiab] OR premature infant*[tiab] OR placental abruption*[tiab] OR abruptio placentae[tiab] OR placenta previa[tiab] OR premature rupture of membrane[tiab] OR perinatal death[tiab] OR preeclampsia[tiab] OR eclampsia*[tiab] OR HELLP syndrome[tiab] OR Toxemia*[tiab] OR EPH Gestosis[tiab] OR EPH Complex[tiab] OR Edema-Proteinuria-Hypertension[tiab] OR Perinatal mortality[tiab])

Limited to last 5 years

Embase

('thyroid gland'/exp OR 'thyroid disease'/exp OR 'thyroid hormone'/exp OR 'thyroid hormone receptor'/exp OR 'thyroid cancer'/exp OR 'thyroid function'/exp OR 'thyroid function test'/exp OR 'antithyroid agent'/exp OR thyroid*:ab,ti,kw OR hypothyroid*:ab,ti,kw OR hyperthyroid*:ab,ti,kw OR dysthyroid*:ab,ti,kw OR (grave* NEAR/1 disease) OR (basedow* NEAR/1 disease) OR (hashimoto* NEAR/1 (struma OR syndrome OR disease)) OR thyrotoxicosis:ab,ti,kw OR hyperthyroxinemia:ab,ti,kw OR myxedema:ab,ti,kw OR thyroiditis:ab,ti,kw OR 'ths deficien*':ab,ti,kw OR calcitonin:ab,ti,kw OR dextrothyroxine:ab,ti,kw OR diiodotyrosine:ab,ti,kw OR monoiodotyrosine:ab,ti,kw OR thyronines:ab,ti,kw OR thyroxine:ab,ti,kw OR triiodothyronine:ab,ti,kw OR thyroidectomy:ab,ti,kw OR thyroidectomies:ab,ti,kw OR 't3 receptor*':ab,ti,kw OR 't4 receptor*':ab,ti,kw OR 'dit receptor*':ab,ti,kw OR 'mit receptor*':ab,ti,kw OR 't3 test*':ab,ti,kw OR 't4 test*':ab,ti,kw OR 'tsh test*':ab,ti,kw OR 'thyroglobulin antibody':ab,ti,kw OR antithyroid*:ab,ti,kw OR 'anti thyroid*':ab,ti,kw OR euthyroid:ab,ti,kw) AND ('female genital system'/exp OR 'gynecologic disease'/exp OR 'menarche'/exp OR 'female genital system function'/exp OR 'parameters concerning the fetus, newborn and pregnancy'/exp OR 'pregnancy disorder'/exp OR reproduction:ab,ti,kw OR reproductive:ab,ti,kw OR sexual:ab,ti,kw OR intercourse:ab,ti,kw OR conception*:ab,ti,kw OR preconception*:ab,ti,kw OR periconception*:ab,ti,kw OR contraceptive*:ab,ti,kw OR uterus:ab,ti,kw OR uteri:ab,ti,kw OR uterine:ab,ti,kw OR ovary:ab,ti,kw OR ovaries:ab,ti,kw OR ovarian:ab,ti,kw OR oocyte*:ab,ti,kw OR oogenesis:ab,ti,kw OR embryo*:ab,ti,kw OR endometri*:ab,ti,kw OR pcos:ab,ti,kw OR pregnan*:ab,ti,kw OR obstetric:ab,ti,kw OR obstetrical:ab,ti,kw OR gynecol*:ab,ti,kw OR antenatal:ab,ti,kw OR prenatal:ab,ti,kw OR childbirth:ab,ti,kw OR 'child

birth':ab,ti,kw OR childbearing:ab,ti,kw OR labor:ab,ti,kw OR labour:ab,ti,kw OR parturition:ab,ti,kw OR 'delivery outcome*':ab,ti,kw OR menarche:ab,ti,kw OR gametogenesis:ab,ti,kw OR ovulat*:ab,ti,kw OR menstrual:ab,ti,kw OR menstruat*:ab,ti,kw OR menopaus*:ab,ti,kw OR premenopaus*:ab,ti,kw OR gestation*:ab,ti,kw OR gravid*:ab,ti,kw OR fertility:ab,ti,kw OR fertile:ab,ti,kw OR infertility:ab,ti,kw OR infertile:ab,ti,kw OR subfertility:ab,ti,kw OR subfertile:ab,ti,kw OR fecundity:ab,ti,kw OR fertilization:ab,ti,kw OR stillbirth*:ab,ti,kw OR stillborn*:ab,ti,kw OR miscarriage*:ab,ti,kw OR abortion*:ab,ti,kw OR 'fetal loss*':ab,ti,kw OR 'fetal death*':ab,ti,kw OR 'low birth weight*':ab,ti,kw OR 'low birthweight*':ab,ti,kw OR 'small for gestational age':ab,ti,kw OR sga:ab,ti,kw OR 'growth retardation':ab,ti,kw OR 'growth restriction':ab,ti,kw OR 'restricted growth':ab,ti,kw OR iugr:ab,ti,kw OR 'preterm birth*':ab,ti,kw OR 'pre-term birth*':ab,ti,kw OR 'premature birth*':ab,ti,kw OR 'premature labor':ab,ti,kw OR 'preterm labor':ab,ti,kw OR 'preterm deliver*':ab,ti,kw OR 'premature deliver*':ab,ti,kw OR 'preterm infant*':ab,ti,kw OR 'premature infant*':ab,ti,kw OR 'placental abruption*':ab,ti,kw OR 'abruptio placentae':ab,ti,kw OR 'placenta previa':ab,ti,kw OR 'premature rupture of membrane':ab,ti,kw OR 'perinatal death':ab,ti,kw OR preeclampsia:ab,ti,kw OR eclampsia*:ab,ti,kw OR 'hellp syndrome':ab,ti,kw OR toxemia*:ab,ti,kw OR 'eph gestosis':ab,ti,kw OR 'eph complex':ab,ti,kw OR 'edema-proteinuria-hypertension':ab,ti,kw OR 'perinatal mortality':ab,ti,kw)

Limited to last 5 years

Web of Science

(TI=(Thyroid* OR goiter OR hyperthyroid* OR hypothyroid* OR dysthyroid* OR (grave* NEAR/1 disease) OR (Basedow* NEAR/1 Disease) OR (Hashimoto* NEAR/1 (Struma OR syndrome OR disease)) OR thyrotoxicosis OR hyperthyroxinemia OR myxedema OR thyroiditis OR “ths deficien*” OR calcitonin OR dextrothyroxine OR diiodotyrosine OR monoiodotyrosine OR thyronines OR thyroxine OR triiodothyronine OR thyroidectomy OR Thyroidectomies OR “t3 receptor*” OR “t4 receptor*” OR “DIT receptor*” OR “MIT receptor*” OR “T3 test*” OR “t4 test*” OR “tsh test*” OR “thyroglobulin antibody” OR antithyroid* OR anti-thyroid* OR euthyroid) OR AB=(Thyroid* OR goiter OR hyperthyroid* OR hypothyroid* OR dysthyroid* OR (grave* NEAR/1 disease) OR (Basedow* NEAR/1 Disease) OR (Hashimoto* NEAR/1 (Struma OR syndrome OR disease)) OR thyrotoxicosis OR hyperthyroxinemia OR myxedema OR thyroiditis OR “ths deficien*” OR calcitonin OR dextrothyroxine OR diiodotyrosine OR monoiodotyrosine OR thyronines OR thyroxine OR triiodothyronine OR thyroidectomy OR Thyroidectomies OR “t3 receptor*” OR “t4 receptor*” OR “DIT receptor*” OR “MIT receptor*” OR “T3 test*” OR “t4 test*” OR “tsh test*” OR “thyroglobulin antibody” OR antithyroid* OR anti-thyroid* OR euthyroid) OR AK=(Thyroid* OR goiter OR hyperthyroid* OR hypothyroid* OR dysthyroid* OR (grave* NEAR/1 disease) OR (Basedow* NEAR/1 Disease) OR (Hashimoto* NEAR/1 (Struma OR syndrome OR disease)) OR thyrotoxicosis OR hyperthyroxinemia OR myxedema OR thyroiditis OR “ths deficien*” OR calcitonin OR dextrothyroxine OR diiodotyrosine OR monoiodotyrosine OR thyronines OR thyroxine OR triiodothyronine OR thyroidectomy OR Thyroidectomies OR “t3 receptor*” OR “t4 receptor*” OR “DIT receptor*” OR “MIT receptor*” OR “T3 test*” OR “t4 test*” OR “tsh test*” OR “thyroglobulin antibody” OR antithyroid* OR anti-thyroid* OR euthyroid)) AND (TI=(reproduction OR reproductive OR sexual OR intercourse OR conception* OR preconception* OR periconception* OR contraceptive* OR uterus OR uteri OR uterine OR ovary OR ovaries OR ovarian OR oocyte* OR oogenesis OR endometri* OR PCOS OR pregnan* OR obstetric OR obstetrical OR gynecol* OR antenatal OR prenatal OR childbirth OR “child birth” OR childbearing OR labor OR labour OR parturition OR “delivery outcome*” OR menarche OR amenorrhea OR gametogenesis OR ovulat* OR menstrual OR menstruat* OR menopaus* OR premenopaus* OR gestation* OR gravid* OR fertility OR fertile OR infertility OR infertile OR subfertility OR subfertile OR

fecundity OR fertilization OR stillbirth* OR stillborn* OR miscarriage* OR abortion* OR “fetal loss*” OR “fetal death*” OR “low birth weight*” OR “low birthweight*” OR “small for gestational age” OR ((“growth retardation” OR “growth restriction” OR “restricted growth”) NEAR/2 (prenatal OR fetal OR foetal OR fetus OR foetus)) OR IUGR OR “preterm birth*” OR “pre-term birth*” OR “premature birth*” OR “premature labor” OR “preterm labor” OR “preterm deliver*” OR “premature deliver*” OR “preterm infant*” OR “premature infant*” OR “placental abruption*” OR “abruptio placentae” OR “placenta previa” OR “premature rupture of membrane” OR “perinatal death” OR preeclampsia OR eclampsia* OR “HELLP syndrome” OR Toxemia* OR “EPH Gestosis” OR “EPH Complex” OR “Edema-Proteinuria-Hypertension” OR “Perinatal mortality”) OR AB=(reproduction OR reproductive OR sexual OR intercourse OR conception* OR preconception* OR periconception* OR contraceptive* OR uterus OR uteri OR uterine OR ovary OR ovaries OR ovarian OR oocyte* OR oogenesis OR endometri* OR PCOS OR pregnan* OR obstetric OR obstetrical OR gynecol* OR antenatal OR prenatal OR childbirth OR “child birth” OR childbearing OR labor OR labour OR parturition OR “delivery outcome*” OR menarche OR amenorrhea OR gametogenesis OR ovulat* OR menstrual OR menstruat* OR menopaus* OR premenopaus* OR gestation* OR gravid* OR fertility OR fertile OR infertility OR infertile OR subfertility OR subfertile OR fecundity OR fertilization OR stillbirth* OR stillborn* OR miscarriage* OR abortion* OR “fetal loss*” OR “fetal death*” OR “low birth weight*” OR “low birthweight*” OR “small for gestational age” OR ((“growth retardation” OR “growth restriction” OR “restricted growth”) NEAR/2 (prenatal OR fetal OR foetal OR fetus OR foetus)) OR IUGR OR “preterm birth*” OR “pre-term birth*” OR “premature birth*” OR “premature labor” OR “preterm labor” OR “preterm deliver*” OR “premature deliver*” OR “preterm infant*” OR “premature infant*” OR “placental abruption*” OR “abruptio placentae” OR “placenta previa” OR “premature rupture of membrane” OR “perinatal death” OR preeclampsia OR eclampsia* OR “HELLP syndrome” OR Toxemia* OR “EPH Gestosis” OR “EPH Complex” OR “Edema-Proteinuria-Hypertension” OR “Perinatal mortality”) OR AK=(reproduction OR reproductive OR sexual OR intercourse OR conception* OR preconception* OR periconception* OR contraceptive* OR uterus OR uteri OR uterine OR ovary OR ovaries OR ovarian OR oocyte* OR oogenesis OR endometri* OR PCOS OR pregnan* OR obstetric OR obstetrical OR gynecol* OR antenatal OR prenatal OR childbirth OR “child birth” OR childbearing OR labor OR labour OR parturition OR “delivery outcome*” OR menarche OR amenorrhea OR gametogenesis OR ovulat* OR menstrual OR menstruat* OR menopaus* OR premenopaus* OR gestation* OR gravid* OR fertility OR fertile OR infertility OR infertile OR subfertility OR subfertile OR fecundity OR fertilization OR stillbirth* OR stillborn* OR miscarriage* OR abortion* OR “fetal loss*” OR “fetal death*” OR “low birth weight*” OR “low birthweight*” OR “small for gestational age” OR ((“growth retardation” OR “growth restriction” OR “restricted growth”) NEAR/2 (prenatal OR fetal OR foetal OR fetus OR foetus)) OR IUGR OR “preterm birth*” OR “pre-term birth*” OR “premature birth*” OR “premature labor” OR “preterm labor” OR “preterm deliver*” OR “premature deliver*” OR “preterm infant*” OR “premature infant*” OR “placental abruption*” OR “abruptio placentae” OR “placenta previa” OR “premature rupture of membrane” OR “perinatal death” OR preeclampsia OR eclampsia* OR “HELLP syndrome” OR Toxemia* OR “EPH Gestosis” OR “EPH Complex” OR “Edema-Proteinuria-Hypertension” OR “Perinatal mortality”))

Limited to last 5 years
